# The transfer function of the LIF model: from white to filtered noise

**DOI:** 10.1186/1471-2202-15-S1-P146

**Published:** 2014-07-21

**Authors:** Jannis Schuecker, Markus Diesmann, Moritz Helias

**Affiliations:** 1 Institute of Neuroscience and Medicine (INM-6) and Institute for Advanced Simulation (IAS-6), Jülich Research Centre and JARA, Jülich, Germany; 2 RIKEN Brain Science Institute, Wako, Saitama, Japan; 3 Medical Faculty, RWTH Aachen University, Germany

## 

**Figure 1:**
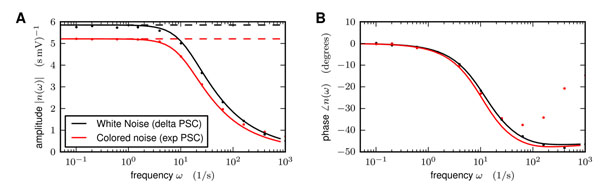
**A** Linear response amplitude for neurons exposed to colored (red) and white (black) noise. Simulations (dots) and analytical results (curves). **B** Phase shift of linear response.

The theory describing correlated activity emerging in recurrent networks relies on the single neuron response to a modulation of its input, i.e. the transfer function. For the leaky integrate-and-fire neuron model exposed to unfiltered synaptic noise the transfer function can be derived analytically [1,2]. In this context the effect of synaptic filtering on the response properties has also been studied intensively at the beginning of the last decade [3,4]. Analytical results were derived in the low as well as in the high frequency limit. The main finding is that the linear response amplitude of model neurons exposed to filtered synaptic noise does not decay to zero in the high frequency limit. A numerical method has also been developed to study the influence of synaptic noise on the response properties [5]. Here we first revisit the transfer function for neuron models without synaptic filtering and simplify the derivation exploiting analogies between the one dimensional Fokker-Planck equation and the quantum harmonic oscillator. We treat the problem of synaptic filtering with short time constants by reducing the corresponding two dimensional Fokker-Planck equation to one dimension with effective boundary conditions [6]. To this end we use the static and dynamic boundary conditions derived earlier by a perturbative treatment of the arising boundary layer problem [4]. Finally we compare the analytical results to direct simulations (Fig.1) and observe that the approximations are valid up to frequencies in the gamma range (60-80 Hz). Deviations are explained by the nature of the approximations.
